# Checking vesicourethral anastomosis for urinary extravasation during radical prostatectomy: is it still necessary in the robotic era? A prospective, randomized case-control study

**DOI:** 10.1007/s00345-024-05177-w

**Published:** 2024-08-22

**Authors:** Bora Ozveren, Nejdet Karsiyakali, Mahir Bulent Ozgen, Levent Turkeri

**Affiliations:** 1 Department of Urology, Acibadem M.A. Aydinlar University, School of Medicine, Altunizade Hospital, Istanbul, Turkey; 2 Department of Urology, Acibadem M.A. Aydinlar University, Altunizade Hospital, Istanbul, Turkey

**Keywords:** Prostate cancer, Robot-assisted radical prostatectomy, Cystogram, Urinary extravasation, Vesicourethral anastomosis

## Abstract

**Purpose:**

This study aims to evaluate the role of intraoperative control of the watertightness of vesicourethral anastomosis extravasation control (VUAEC) in predicting vesicourethral anastomosis (VUA) healing and early postoperative outcomes in patients undergoing robot-assisted radical prostatectomy (RARP).

**Methods:**

100 patients who underwent RARP between October 2020 and May 2023 were consecutively included in the study. Preoperatively, the patients were randomized to undergo VUAEC (**Group-A**) or not (**Group-B**). Patients in **Group-A** were evaluated in 2 subgroups: those with no extravasation observed during VUAEC (**Group-A1**; *n* = 31 (62%)) and those with extravasation (**Group-A2**; *n* = 19 (38%)). On the 8th post-operative day, a gravity cystogram (GC) was performed on all patients to assess VUA healing.

**Results:**

There was no statistically significant difference between the groups in terms of clinical features, drain removal time, length of hospital stay, extravasation on GC, catheter removal time and postoperative complications (*p* > 0.05, for each). There was also no statistically significant difference between the subgroups in terms of drain removal time, length of hospital stays, catheter removal time (*p* > 0.05, for each). In **Group-A2**, urinary extravasation on GC was found in a greater percentage, but the difference remained statistically insignificant (*p* = 0.082).

**Conclusions:**

Performing intraoperative VUAEC did not have a significant role in the prediction of VUA healing and early postoperative outcomes in patients undergoing RARP. The current study did not identify a substantial clinical benefit of routine intraoperative VUAEC.

## Introduction

The suturing of the vesicourethral anastomosis (VUA) can be a formidable task regardless of surgical approach, especially when exposure is inadequate. Flawed suturing can lead to postoperative urinary extravasation, resulting in various complications including persistent drainage, prolonged catheterization, extended hospital stays, infections, urinomas, incontinence, and anastomotic strictures [1]. Anastomotic leakage may furthermore cause ileus, particularly in transperitoneal laparoscopic and robotic surgeries.

Achieving a watertight VUA appears imperative for preventing complications and attempting early removal of the catheter. Generally, VUA integrity is assessed intraoperatively by bladder fill/flush using sterile saline (VUA extravasation control [VUAEC]) via the urethral catheter after completion of anastomosis. To date, numerous innovations and techniques have been employed for anastomotic integrity, including knotting techniques (interrupted vs. running), bladder neck reconstruction methods, innovative suture materials (barbed sutures), improved surgical exposure and instrumentations (open vs. laparoscopic vs. robotic) [2–8]. The practice of intraoperative VUAEC, though not in a standardized fashion, has traditionally been utilized as a safe and reliable method for assessing anastomotic integrity.

It is known that urine extravasation can be observed during the postoperative cystography even in patients who have a watertight VUA proven by VUAEC [9]. To the best of our knowledge, the literature lacks verification of the impact of this practice on VUA healing and early postoperative outcomes. The present study aimed to explore the relationship between intraoperative VUAEC test findings and early postoperative outcomes in patients undergoing robot-assisted radical prostatectomy (RARP).

## Materials and methods

### Study population and surgical approach

Eligibility criteria in this prospective, randomized case-control study included a hundred consecutive patients diagnosed with localized prostate cancer who underwent RARP at a single center between October 2020 and May 2023. The study protocol was approved by the local Institutional Ethics Committee (IRB No: 2020-27/22). Written informed consent was obtained from all patients. Patients with a history of pelvic surgery, radiotherapy, and/or prior transurethral surgery were excluded.

Patients were preoperatively assigned randomly in a 1:1 ratio to either undergo VUAEC (**Group-A**) or not (**Group-B**) using the *Randomizer for Clinical Trial Lite©* version 2.3 application.

Detailed demographic and preoperative clinical characteristics, perioperative surgical parameters, including surgery duration, console time, estimated blood loss, drainage amount, drain removal time, length of hospital stay, gravity cystogram (GC) findings, catheter removal time, and postoperative complications as per the Clavien-Dindo (C-D) classification [10] were recorded.

A highly experienced single surgeon (L.T.) performed all RARPs using DaVinci^®^Xi Surgical Systems (Intuitive Surgical, Sunnyvale, CA, USA). Anterior (a) RARP procedures followed the description by Rocco et al. [11], while retzius-sparing (rs) RARP procedures were conducted as per Galfano’s technique [12]. VUAs were created using bidirectional 3 –0 V-Loc™ barbed sutures as per the van Velthoven’s technique [2]. The surgeon remained blinded to the randomization until completing the final VUA knot during RARP.

In this randomized case-control study, a standard bladder filling method was applied. In **Group-A**, 120 mL of sterile physiologic saline was introduced via the urethral catheter for VUAEC. Any urinary leakage from the VUA was recorded as a positive result. Patients in **Group-A** were further divided into two subgroups: those with no extravasation during VUAEC (**Group-A1**) and those with visible extravasation (**Group-A2**). No attempt was made to fix the leakage site or restore the VUA when a leak was noted. In **Group-B**, the VUA was performed with the same technique and the patients did not undergo an intraoperative saline flush test regardless of any intraoperative finding.

### Gravity cystograms

On the 8th postoperative day, VUA healing was assessed by GC following a prophylactic fosfomycin trometamol administration. The bladder was filled via the 18-French silicone urethral catheter with contrast solution (150 mL of sterile saline + 100 mL of amidotrizoate (Urografin^®^)). Anastomotic extravasation was checked by anteroposterior and oblique fluoroscopic imaging at full and empty bladder phases. The catheter was removed if no extravasation was observed. In cases of minimal extravasation, the catheter was removed 2–3 days later without repeating the GC. When significant extravasation was observed, the GC was repeated one week later. The catheter removal time was noted for each patient.

### Statistical analysis

Statistical analysis was conducted using the Statistical Package for the Social Sciences (SPSS) version 22.0 software (IBM Corp., Armonk, NY, USA). The normality of data for quantitative variables was assessed using the Shapiro-Wilk test. Continuous variables were expressed in median and interquartile range, while categorical variables were presented as the number and percentage. The Pearson chi-square, Fisher’s exact, and Mann- Whitney U tests were employed as appropriate. A two-sided *p*-value of < 0.05 was considered statistically significant. Since the anastomotic leakage after RARP ranged from 0.1 to 15.4% [13, 14], the standard effect size was calculated as 0.57 with a 5% α-risk and 80% power. Hence, it was determined that at least 48 cases were needed for each group.

## Results

There was no significant difference in the demographic features, clinical parameters, and frequency of risk factors between the groups (Table-1).

**Table 1:** Comparisons of the groups in terms of demographic and preoperative clinical parameters

		Group										
		Case (Group-A) (*n* = 50, 50%)					Control (Group-B) (*n* = 50, 50%)					
		median (IQR)			*n*, %		median (IQR)			*n*, %		*p*-value
Age (year)		66 (62–69)					66 (62–68)					^a^0.548
BMI (kg/m^2^)		28.10 (26.20–30.50)					27.00 (24.40–29.10)					^a^0.063
Diabetes Mellitus (yes)					7 (14.0%)					9 (18,0%)		^b^0.585
Hypertension (yes)					30 (60.0%)					30 (60,0%)		^b^1.000
Coronary Artery Disease (yes)					11 (22.0%)					9 (18,0%)		^b^0.617
Chronic Obstructive Pulmonary Disease (yes)					4 (8.0%)					1 (2,0%)		^c^0.362
ASA Class	ASA-1				10 (20.0%)					8 (16,0%)		^c^0.845
	ASA-2				39 (78.0%)					40 (80,0%)		
	ASA-3				1 (2.0%)					2 (4,0%)		
Preoperative PSA Level (ng/mL)		6.92 (5.30–9.40)					7.05 (4.82–9.48)					^a^0.639
Prostate Volume (mL)		47.35 (34.65–69.00)					40.12 (31.00–52.62)					^a^0.085
Digital Rectal Examination (suspicious)					17 (34.0%)					24 (48,0%)		^b^0.155
Smoking History	Never				18 (36.0%)					22 (44,0%)		^b^0.260
	Quit				24 (48.0%)					25 (50,0%)		
	Smoker				8 (16.0%)					3 (6,0%)		
Cigarette Package Year (year)		7 (0–20)					5 (0–25)					^a^0.540

^a^Mann-Whitney-U test, ^b^Chi-square test, ^c^Fishers’s exact test

IQR: Interquartile range, BMI: Body mass index, ASA: American Society of Anesthesiologists, PSA: Prostate specific antigen

rsRARP and aRARP were performed in 59% and 41% of patients, respectively. Median operative and console time were 251 (209–285) and 196 (161–227) minutes, respectively. The estimated blood loss was 50 (50–95) mL. No intraoperative complication was observed. The median drain removal time was 2 (2–2) days. The median volume of total and average daily drainage was 125 (60–245) and 65 (36–120) mL, respectively. Median length of hospital stay was 3 (2–3) days. Extravasation on GC was seen in 30 (30%) patients. The median catheter removal time was 8 (8–14) days.

C-D-I, -II, -IIIA, and -IIIB complications were seen in 4 (4%) [1 haematuria, 3 early re-catheterizations after catheter removal], 5 (%5) [4 paralytic ileuses, 1 blood transfusion], 1 (1%) [transperineal injection of Tisseel fibrin sealant into the anastomotic gap via transrectal ultrasonographic guidance under local anesthesia, for prolonged leakage on the postoperative 4th week], and 1 (1%) [spigelian hernia repair under general anesthesia] patients, respectively. The median follow-up time was 23 (16–34) months and no VUA stricture was observed.

There was no statistically significant difference between **Group-A** and **Group-B** in terms of intra- and post-operative measures, urine extravasation on the GC, catheter removal time, and postoperative complications (*p* > 0.05, for each) (Table-1).

**Table 2:** Comparisons of the groups in terms of peroperative parameters

		Group				
		Case (Group-A) (*n* = 50, 50%)		Control (Group-B) (*n* = 50, 50%)		
		*n*, %	median (IQR)	*n*, %	median (IQR)	*p*-value
Surgical Approach	Retzius-Sparing	25 (50.0%)		34 (68.0%)		^a^0.067
	Standard (Anterior)	25 (50.0%)		16 (32.0%)		
Operation Duration (min)			251 (219–289)		257 (205–285)	^b^0,732
Console Time (min)			196 (165–229)		193 (159–224)	^b^0,863
Estimated Blood Loss (mL)			50 (50–100)		50 (50–50)	^b^0,171
Drain Removal Time (day)			2 (2–2)		2 (2–2)	^b^0,946
Total Drainage (mL)			110.00 (60.00–220.00)		155.00 (60.00–250.00)	^b^0,521
Length of Hospital Stay (day)			3 (2–3)		3 (2–3)	^b^0,891
Urinary Extravasation on the Gravity Cystogram (yes)		14 (28.0%)		16 (32.0%)		^a^0,663
Catheter Removal Time (day)			8 (8–14)		8 (8–14)	^b^0,770
Postoperative Complication (Clavien-Dindo)	No	45 (90.0%)		44 (88.0%)		^c^0,320
	Clavien-Dindo-I	3 (6.0%)		1 (2.0%)		
	Clavien-Dindo-II	1 (2.0%)		4 (8.0%)		
	Clavien-Dindo-IIIA	0 (0.0%)		1 (2.0%)		
	Clavien-Dindo-IIIB	1 (2.0%)		0 (0.0%)		

^a^Chi-square test, ^b^Mann-Whitney-U test, ^c^Fishers’s exact test

IQR: Interquartile range

In **Group-A** (*n* = 50), VUAEC revealed no extravasation in 31 (62%) patients (**Group-A1)**, whereas any amount of leakage was observed in 19 (38%) patients (**Group-A2**). There was no statistically significant difference between the subgroups in terms of drain removal time, drainage amounts, length of hospital stay, urine extravasation on the GC, catheter removal time and postoperative complications (*p* > 0.05, for each) (Table-1).

**Table 3:** Comparisons of the subgroups according to urinary extravasation on VUAEC in terms of postoperative clinical parameters

		Extravasation on VUAEC				
		No (Group-A1) (*n* = 31, 62.0%)		Yes (Group-A2) (*n* = 19, 38%)		
		median (IQR)	*n*, %	median (IQR)	*n*, %	*p*-value
Drain Removal Time (day)		2 (2–2)		2 (2–2)		^a^0.649
Total Drainage (mL)		120.00 (60.00–220.00)		90.00 (60.00–250.00)		^a^0.787
Length of Hospital Stay (day)		3 (2–3)		3 (2–3)		^a^0.422
Urinary Extravasation on the Gravity Cystogram (yes)			6 (19.4%)		8 (42.1%)	^b^0.082
Catheter Removal Time (day)		8 (8–10)		8 (8–15)		^a^0.135
Postoperative Complication (Clavien-Dindo)	No		27 (87.1%)		18 (94.7%)	^c^1.000
	Clavien-Dindo-I		2 (6.5%)		1 (5.3%)	
	Clavien-Dindo-II		1 (3.2%)		0 (0.0%)	
	Clavien-Dindo-IIIA		0 (0.0%)		0 (0.0%)	
	Clavien-Dindo-IIIB		1 (3.2%)		0 (0.0%)	

VUAEC: vesicourethral anastomosis extravasation control

^a^Mann-Whitney-U test, ^b^Chi-square test, ^c^Fishers’s exact test

IQR: Interquartile range

## Discussion

Since prolonged catheterization after radical prostatectomy carries several risks such as urinary tract infections, anastomotic strictures, and impaired quality of life, investigations have attempted to determine the shortest postoperative catheterization time [9, 15–18]. Currently, urethral catheters are usually removed on postoperative days 7–10 [9, 19]. Formerly, the common practice was removing the Foley catheter 3 weeks after radical retropubic prostatectomy after being evaluated by GC [20]. Since then, several technical developments have been aimed at achieving watertight anastomosis [2–4, 8, 15, 16, 20, 21]. While few studies suggested that catheters may be removed as early as 3–4 days albeit with a higher risk of acute urinary retention, most of the research confirmed that urethral catheters can safely be removed after 2–3 weeks without any concern of urinary retention, urinoma, pelvic abscess, or anastomotic stricture formation [15, 16, 21]. Furthermore, Leibovitch et al. reported that the presence of minimal urinary extravasation at the VUA site in GC did not affect the clinical outcomes [22]. Although a cystographic evaluation is usually recommended if an early de-catheterization is planned, some authors suggest that it may be unnecessary if the catheterization period is 14 to 21 days in patients with no postoperative prolonged or excessive drainage [22]. The incidence of anastomotic leakage after RARP has previously been reported in approximately 0.1–15.4% of patients [13, 14]. In the present study, we observed anastomotic leakage in about 3 of 10 patients on the 8th day post-operatively in both the test and control groups. On the other hand, subgroup analysis revealed that patients who presumably had a watertight VUA based on intraoperative VUAEC findings had a significantly lower rate of anastomotic leakage at postoperative day 8 (19.4% vs. 42.1%). Therefore, in cases where VUAEC showed leakage, we advocate that GC should certainly be performed if catheter removal is planned earlier than 10–14 days after RARP.

The significance of the intraoperative VUAEC test in the prediction of anastomotic integrity or healing has so far been empirical. The extravasation of saline during this test is customarily regarded as a sign of failure of the immediate integrity of anastomosis, though available literature lacks specific information on the technique, such as the speed or the amount of filling. Furthermore, in most of the studies, the methodology is not clear whether this test serves any role in restoring the VUA. In this randomized case-control study, we applied a standard bladder filling method, and any amount of fluid observed was regarded as an anastomotic leak. However, no attempt was made to fix the leakage site or restore the VUA when a leak was noted. The technique of anastomosis was practically consistent as all RARPs were carried out by the same surgeon.

The current findings demonstrate no difference in postoperative GC findings, catheter removal time, and complications between groups with or without intraoperative VUAEC testing during robotic surgery. Nevertheless, the most obvious finding to emerge from the subgroup analysis is that the urinary leakage ratio on GC was higher in patients who had an intraoperative anastomotic leak. Therefore, we postulate that the demonstration of leakage intraoperatively may predict hampered healing at the VUA. Hence, VUAEC practice may serve in deciding on the quality of bladder neck reconstruction or suturing of VUA. An intraoperative anastomotic leak should prompt the surgeon to re-create or reinforce the anastomosis to decrease the risk of VUA healing.

Specific risk factors involving a history of ischemic heart disease, TUR-P, obesity, very large prostate size, increased intraoperative bleeding, urinary tract infections, and impaired visibility/difficult anastomosis may conceivably lead to persistent leakage at the VUA postoperatively [14, 23–25]. Despite some contradictory results, none of these probable risk factors were found reliably predictive of delayed VUA healing that may complicate the early and late postoperative period. In this study, we did not observe the impact of any demographic or clinical factors on anastomotic leakage. Many authors proposed that the quality of VUA in RARP remains the principal factor associated with decreased morbidity [3, 4, 26]. Similarly, in our cohort, none of the patient comorbidities or characteristics was identifiable as a factor for persistent urinary leakage. Blood loss was minimal, while TUR-P history was one of the exclusion criteria. Nevertheless, regardless of the presence of plausible risk factors, a difficult anastomosis has been shown as an independent factor for VUA leakage. Thus, in such cases, VUAEC may be worthwhile in assessing the quality of VUA. However, we do not think that VUAEC plays a pivotal role in predicting postoperative GC findings according to our findings.

The major strength of the present study is that this is the first randomized case-control study that assesses the effect of VUAEC on VUA healing and early postoperative outcomes exclusively in patients undergoing RARP. This study has several limitations. Firstly, it is a single-center study with a single surgeon performing all RARPs. Secondly, the cohort involved two separate RARP approaches albeit in even distribution among groups and subgroups. As the early continence rates may favor rsRARP in comparison to aRARP, the different surgical approaches may conceivably have an impact on VUA healing as well. Nevertheless, the urinary extravasation rates on GC remained similar in both surgical approaches (25.4% in rsRARP vs. 36.6% in aRARP, *p* = 0.231).

## Conclusions

Despite the increasing experience and technical modifications, the outcomes still fall short of granting a consistent quality of VUA in the robotic surgery era. In this regard, the current prospective randomized case-control study fails to identify a substantial clinical benefit of routine intraoperative VUAEC. However, our findings reveal that the intraoperative demonstration of anastomotic leakage predicts delayed healing, and therefore, in this subset of patients, GC may be beneficial if early catheter removal is considered.

## Data Availability

All the data supporting our findings is contained in the manuscript. The datasets used and/or analysed in the current study is available from the corresponding author on reasonable request.

## Abbreviations and Acronyms

GC: Gravity Cystogram
RARP: Robot-Assisted Radical Prostatectomy
VUA: Vesicourethral Anastomosis
VUAEC: Vesicourethral Anastomosis Extravasation Control
